# PTBP1: A Key Hub and a Potential Therapeutic Target in High-Grade Glioma

**DOI:** 10.3390/ijms27219874

**Published:** 2026-11-04

**Authors:** Cunyu Li, Shuang Hao

**Affiliations:** College of Life and Health Sciences, Northeastern University, Shenyang 110169, China

**Keywords:** high-grade glioma, PTBP1, RNA-binding protein, biomarker

## Abstract

High-grade gliomas (HGGs) are lethal primary brain tumors with poor prognosis despite multimodal therapy. Polypyrimidine tract-binding protein 1 (PTBP1) is an RNA-binding protein that regulates alternative splicing, mRNA stability, and translation. In HGGs, PTBP1 is overexpressed and correlates with tumor grade, IDH mutation status, and patient survival, supporting it as a candidate prognostic biomarker and therapeutic target. Structurally, PTBP1 contains four RNA recognition motifs connected by flexible linkers, conferring conformational plasticity for diverse RNA interactions. In glioma cells, upstream regulators—including non-coding RNAs and post-translational modifications—dysregulate PTBP1, converting it from a physiological regulator into a predominantly oncogenic driver. Multiple lines of evidence suggest that PTBP1 drives malignancy through three interconnected mechanisms: oncogenic splicing switching, stabilization of oncogenic mRNAs, and IRES-dependent translation. Although no selective, direct small-molecule inhibitor of PTBP1 has yet been clinically validated, repurposed compounds (e.g., venetoclax) and indirect modulators have shown preliminary antitumor activity in preclinical models. However, direct target engagement, in vivo efficacy, and blood–brain barrier penetration remain insufficiently established. This review summarizes PTBP1’s structure, upstream networks, downstream mechanisms, and therapeutic potential, highlighting the need for further validation of PTBP1-centered strategies prior to clinical translation.

## 1. Introduction

According to the 2021 WHO classification, adult-type diffuse gliomas are defined by integrated histological and molecular criteria and comprise three main entities: astrocytoma, IDH-mutant (CNS WHO grade 2–4); oligodendroglioma, IDH-mutant and 1p/19q-codeleted (CNS WHO grade 2–3); and glioblastoma, IDH-wildtype (CNS WHO grade 4). High-grade gliomas (HGGs), defined here as CNS WHO grade 3 and 4 adult-type diffuse gliomas, include IDH-wildtype glioblastoma (GBM)—the most common and aggressive primary malignant CNS tumor—as well as IDH-mutant high-grade astrocytoma and oligodendroglioma [1,2,3]. These tumors exhibit obvious intratumoral heterogeneity, therapeutic resistance, diffuse invasiveness, and complex immunosuppression [4,5,6,7]. Despite maximal safe resection followed by radiotherapy and temozolomide chemotherapy, patient prognosis remains poor, with 5-year survival rates under 5% for IDH wild-type GBM patients, and a variable prognosis for those with IDH-mutant high-grade gliomas [8,9,10]. Novel therapeutic strategies are thus urgently needed.

RNA-binding proteins that govern post-transcriptional networks are emerging as promising therapeutic targets in glioma [11,12,13,14,15]. Polypyrimidine tract-binding protein 1 (PTBP1) binds to polypyrimidine tracts in target pre-mRNAs [16] and thereby modulates alternative splicing, mRNA stability and translation [17,18,19]. PTBP1 is overexpressed in various cancers, including colorectal cancer and gastric cancer [20,21] (Table 1), and its upregulation correlates with poor patient survival. In normal brain tissue, PTBP1 is barely detectable in mature neurons [22,23], whereas it is overexpressed in 52.7% of malignant gliomas [24]. Moreover, PTBP1 expression levels correlate with glioma grade, proliferation index, and IDH-wildtype status. In retrospective cohorts, PTBP1 expression has been reported to independently predict overall survival after adjustment for clinicopathological factors (e.g., age, tumor grade, and IDH status), supporting its evaluation as a candidate prognostic biomarker [24].

This review systematically summarizes the biological functions of PTBP1 in glioma, covering its structural basis, upstream regulatory network (including non-coding RNAs and post-translational modifications), and downstream mechanisms—alternative splicing, mRNA stability and translational regulation—by which PTBP1 drives tumorigenesis and progression. We also discuss the therapeutic opportunities and remaining challenges.

Although several recent reviews have summarized the role of PTBP1 in cancer, for example, Ji et al. [17] provided a broad pan-cancer overview of its regulation and therapeutic potential, the present review differs in three key aspects. First, it focuses specifically on high-grade glioma, integrating glioma-specific evidence across PTBP1 structure, upstream non-coding RNA and post-translational regulation, and downstream splicing, mRNA stability, and translation. Second, it critically separates glioma-specific evidence from findings obtained in other cancer types, with the latter presented only as comparative context in Table 1. Third, it provides an evidence-level framework that distinguishes cell lines, patient-derived animal models, and clinical findings, while presenting therapeutic candidates strictly as preclinical rather than clinically validated. These features are intended to complement, rather than duplicate, existing pan-cancer reviews.

## 2. Literature Search and Selection Criteria

This review was based on a non-systematic literature search of PubMed, Web of Science, and Scopus, covering publications from May 2004 to May 2026. Search terms combined “PTBP1” (and its synonyms such as “polypyrimidine tract-binding protein 1” and “hnRNP I”) with terms related to glioma (“glioma”, “glioblastoma”, “high-grade glioma”), RNA regulation (“alternative splicing”, “mRNA stability”, “IRES”, “non-coding RNA”, “microRNA”, “lncRNA”, “circRNA”), and PTBP1 modifications or targeting (“lactylation”, “SUMOylation”, “ubiquitination”, “therapeutic target”). Studies were prioritized if they reported original experimental evidence on PTBP1 regulation, function, or targeting in glioma. Findings from other cancer types were included only to provide mechanistic context and are explicitly identified as such in the text and tables.

## 3. Structure and Function of PTBP1

**Figure 1:**
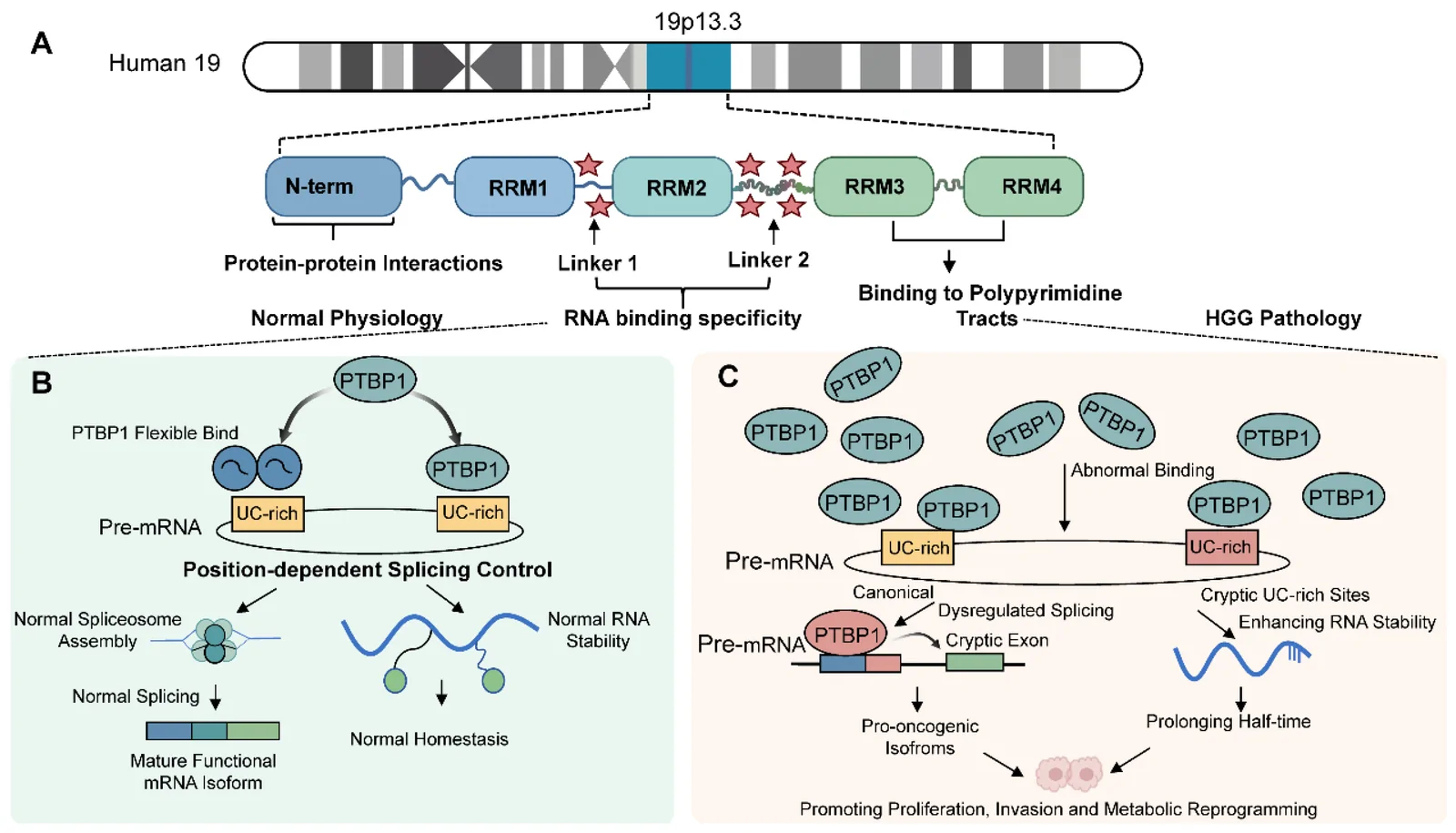
Structural features of PTBP1 and its regulatory mechanisms in high-grade glioma (HGG). (**A**)** Domain architecture.** Human PTBP1 is encoded at the 19p13.3 locus. Its N-terminal domain mediates protein–protein interactions, while four RNA recognition motifs (RRM1–RRM4) are connected by flexible linkers (linkers 1 and 2). RRM1 and RRM2 dictate RNA binding specificity and affinity, whereas RRM3 and RRM4 engage polypyrimidine tracts. The linkers additionally harbor key sites for post-translational modifications (PTMs). (**B**) **Physiological state.** Monomeric PTBP1 bridges distal UC-rich motifs to form an RNA loop. Linker plasticity is necessary for position-dependent splicing and to maintain RNA stability and cellular homeostasis. (**C**) **Pathological state in HGG.** Aberrant PTBP1 overexpression drives the ectopic recognition and bridging of cryptic pre-mRNA sites. This dual action induces the transcription of oncogenic splicing isoforms and stabilizes the selected target mRNAs, increasing their half-lives. These concerted effects ultimately lead to proliferation, invasion and metabolic changes in the tumor cell. Direct promotion or activation of a process is shown as solid arrows (➔) if this has been tested in experiments.

PTBP1 maps to the chromosome 19p13.3 locus and encodes a protein with an N-terminal domain and four RNA recognition motifs (RRMs) [40,41,42] (Figure 1A). The N-terminal domain primarily mediates protein–protein interactions, while the four RRMs are connected by flexible linkers (linker 1 and linker 2) [43,44]. RRM1 and RRM2 contribute substantially to RNA binding specificity and affinity, whereas RRM3 and RRM4 are primarily responsible for high-affinity binding to polypyrimidine tracts [42,43]. Each RRM adopts a conserved β-sheet scaffold that forms the core interface for direct RNA base recognition and binding [45].

Under physiological reducing conditions of the cell, PTBP1 exists as a monomer [46] (Figure 1B). A single monomeric PTBP1 molecule recognizes and binds UC-rich motifs (e.g., UCUU, CUCUCU), thereby bridging distal sites on pre-mRNA to form RNA loops [47,48]. The flexible linkers between RRMs confer conformational plasticity, allowing the monomeric PTBP1 to adopt distinct binding modes in response to the distribution of UC motifs on pre-mRNA [49]. This structural flexibility modulates spliceosome activity and yields position-dependent splicing outcomes [50,51]. Moreover, these linkers are closely linked to post-translational modifications (PTMs) of PTBP1, including lactylation and ubiquitination [20,25,50,52] (Figure 1). This conformational plasticity allows monomeric PTBP1 to govern pre-mRNA splicing and mRNA stability [40].

In HGGs, aberrant overexpression of PTBP1 has been reported to bridge distant RNA-binding sites on pre-mRNA (Figure 1C), leading to abnormally stable binding and regulation of splice sites that are not recognized in normal cells, such as USP5 exon 15 [53]. Moreover, diverse PTMs including lactylation, ubiquitination, and SUMOylation synergize with PTBP1 overexpression to drive a pathological shift from selective physiological regulation to sustained aberrant activation. This shift promotes oncogenic isoform production of key genes, including Brahma/SWI2-related gene 1 (BRG1)-associated factor 45d (BAF45d), ubiquitin-specific peptidase 5 (USP5), and Intersectin 1 (ITSN1), through alternative splicing [26,27,53]. Concurrently, PTBP1 directly binds and stabilizes target mRNAs such as 6-phosphofructo-2-kinase/fructose-2,6-biphosphatase 4 (PFKFB4) and procollagen-lysine, 2-oxoglutarate 5-dioxygenase 3 (PLOD3), significantly prolonging their half-times [25,54,55]. Together, these mechanisms cooperatively drive tumor proliferation, invasion, and metabolic reprogramming (Figure 1).

## 4. Upstream Regulatory Networks of PTBP1

The expression and activity of PTBP1 are regulated in multiple ways. This section summarizes the upstream regulatory networks comprising non-coding RNAs and post-translational modifications, which modulate the abundance and function of PTBP1 in glioma.

### 4.1. Non-Coding RNA-Mediated Regulation of PTBP1

**Figure 2:**
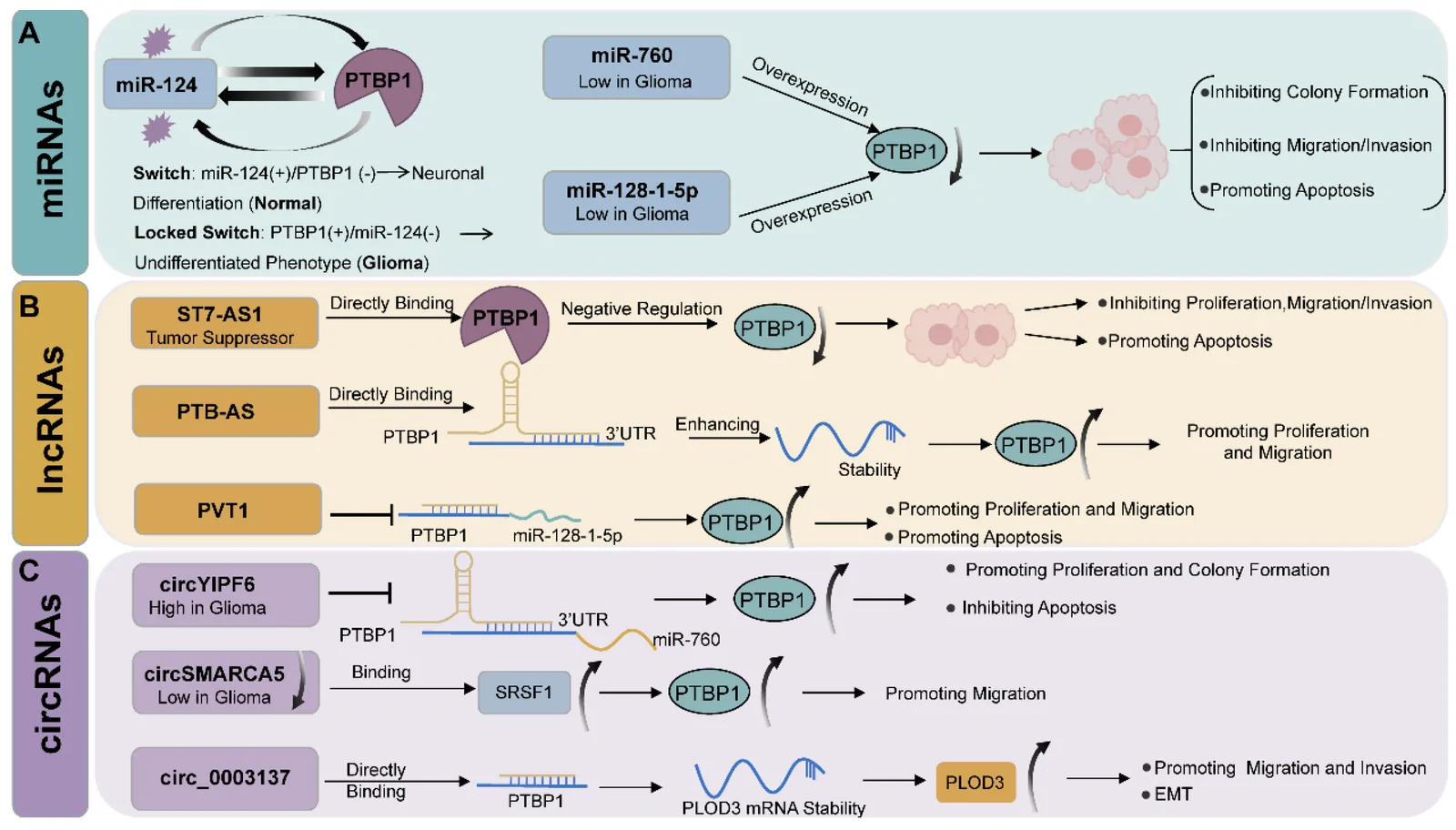
Non-coding RNA-mediated regulation of PTBP1 in glioma. (**A**) **miRNAs.** The miR-124/PTBP1 axis is a bistable switch that promotes neuronal differentiation physiologically but remains locked in a PTBP1-high/miR-124-low state in glioma. miR-760 and miR-128-1-5p are downregulated in glioma and directly repress PTBP1. Silencing these miRNAs relieves PTBP1 repression and promotes tumor progression, whereas overexpressing miR-760 and miR-128-1-5p suppresses PTBP1, inhibiting colony formation and migration/invasion while promoting apoptosis. (**B**) **lncRNAs.** ST7-AS1 directly binds to PTBP1, which leads to the degradation of PTBP1. PTB-AS is associated with mRNA 3’UTR of PTBP1, thus increasing the stability of the mRNA PTBP1 transcript. PVT1 acts as a ceRNA, which binds miR-128-1-5p and prevents it from suppressing PTBP1. (**C**) **circRNAs.** circYIPF6 sponges miR-760 to elevate PTBP1, and circSMARCA5 modulates it indirectly via SRSF1. circ_0003137 directly stabilizes PLOD3 mRNA to drive EMT and invasion. Together, these diverse non-coding RNAs form a complex regulatory network that fine-tunes PTBP1 expression to shape glioma malignancy. Direct promotion or activation (➔), inhibition or repression (⊣) are indicated by solid lines, respectively.

PTBP1 expression in glioma is regulated by diverse non-coding RNAs (Table 2 and Figure 2), including microRNAs (miRNAs), long non-coding RNAs (lncRNAs), and circular RNAs (circRNAs).

**miRNAs:** miRNAs directly bind the 3′ untranslated region (3′UTR) of PTBP1 mRNA, inducing translational repression and transcript degradation [61,62]. Within this network, the neuron-specific miR-124 serves as a key repressor of PTBP1. During normal neuronal differentiation, miR-124 directly represses PTBP1 expression, while PTBP1 in turn blocks miR-124 precursor processing. This mutual inhibition forms a double-negative feedback loop that operates as a bistable switch [56,61,63]. In high-grade gliomas, this bistable switch gets lost, shifting toward a pathological state characterized by PTBP1 overexpression and miR-124 depletion. This locked state can directly promote proliferation, contribute to therapeutic resistance and enforce an undifferentiated tumor phenotype [28,56] (Figure 2A). Other miRNAs also play roles in the regulation of PTBP1 other than miR-124. Depletion of miR-760 causes accumulation of PTBP1. On the other hand, its restoration suppresses proliferation, colony formation, and glycolytic metabolism, along with inducing apoptosis [58]. Likewise, miR-128-1-5p was also significantly downregulated in high-grade gliomas. Restoring its expression silences PTBP1, which, in turn, curtails glioma cell proliferation, migration, and invasion, while triggering DNA damage and cell cycle arrest, ultimately impairing xenograft tumor growth in vivo [57] (Figure 2A).

**lncRNAs:** lncRNAs control PTBP1 expression in glioma through distinct mechanisms. Directly binding to PTBP1 locks it for post-transcriptional degradation by the tumor suppressor ST7-AS1, consequently suppressing the Wnt/β-catenin signaling pathway [59] (Figure 2B). In addition, ST7-AS1 can synergistically inhibit the proliferation, migration and invasion of U251 glioma cells, along with induction of apoptosis in a positive feedback loop with p53 [59]. The antisense lncRNA PTB-AS, which is reverse complementary to the 3′UTR of the PTBP1 mRNA, directly binds to the transcript to recruit the staphylococcal nuclease domain-containing 1 (SND1) complex and, at the same time, masks the miR-9 binding site (Figure 2B). This interaction leads to an increase in mRNA stability and an increase in PTBP1 expression that results in growth and migration of U87 intracranial xenografts [29]. In contrast, plasmacytoma variant translocation 1 (PVT1) functions as a competing endogenous RNA (ceRNA) by sponging miR-128-1-5p (Figure 2B). This sequestration derepresses PTBP1, elevating its expression and driving proliferation and migration while conferring apoptotic resistance in U87 and U251 glioma cells [57].

**circRNAs:** circRNAs regulate PTBP1 expression by competitively binding miRNAs or directly interacting with RNA-binding proteins, thereby influencing glioma progression. Lei et al. reported that circYIPF6 is significantly upregulated in glioma tissues and cell lines (U251, LN229). By sponging miR-760, circYIPF6 relieves the miRNA-mediated repression of PTBP1, which drives enhanced proliferation, colony formation, and glycolysis alongside reduced apoptosis [58] (Figure 2C). Barbagallo et al. found that circSMARCA5 is markedly downregulated in glioblastoma tissues and U87MG cells, and its overexpression inhibits U87MG cell migration. Mechanistically, circSMARCA5 binds the splicing factor SRSF1 and affects SRSF3 pre-mRNA alternative splicing. Loss of circSMARCA5 in GBM releases SRSF1, which alters SRSF3 pre-mRNA splicing [60]. Because SRSF3 interacts with PTBP1, it is hypothesized that the downregulation of circSMARCA5 indirectly leads to the upregulation or activation of PTBP1, potentially driving glioma cell migration (Figure 2C). Additionally, in the glioma microenvironment, hypoxia drives the polarization of tumor-associated macrophages (TAMs) toward an M2-like phenotype in a HIF1α-dependent manner. These M2-like TAMs then deliver circ_0003137 to glioblastoma cells via extracellular vesicles (EVs). In U251 and A172 glioma cells, circ_0003137 directly binds to PTBP1, enhancing PLOD3 mRNA stability and promoting epithelial–mesenchymal transition (EMT), migration, and invasion [55] (Figure 2C).

In summary, non-coding RNAs modulate PTBP1 expression through distinct mechanisms, leading to the regulation of glioma cell proliferation, invasion, and drug resistance, ultimately influencing the initiation and progression of glioma.

### 4.2. Post-Translational Modifications of PTBP1

**Figure 3:**
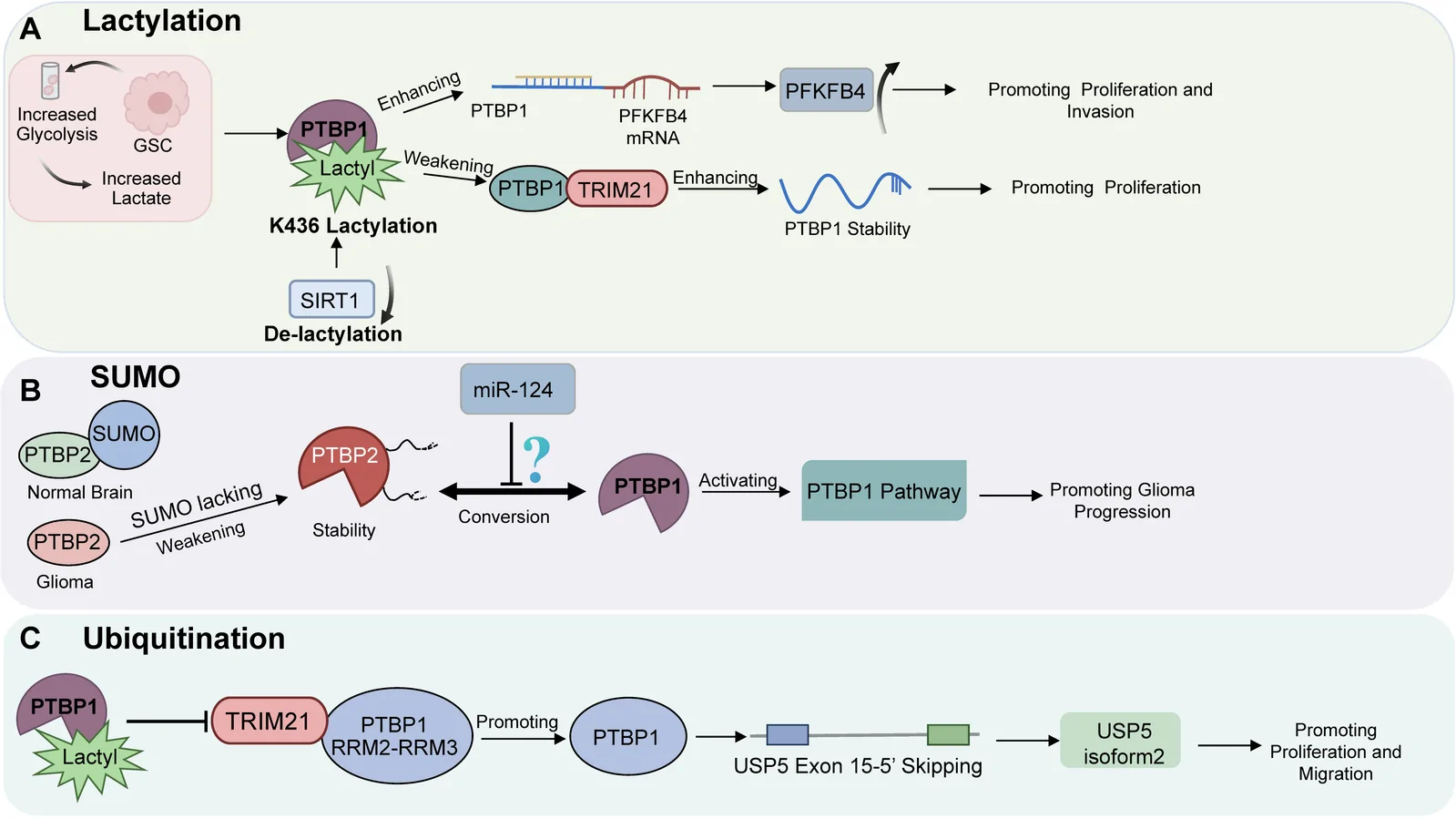
Post-translational modifications (PTMs) regulate PTBP1 function and glioma progression. (**A**) **Lactylation.** Glycolysis-derived lactate in glioma stem cells (GSCs) drives K436 lactylation of PTBP1. This change will increase PFKFB4 mRNA binding and stability while weakening TRIM21-mediated ubiquitination, thus promoting proliferation and invasion. K436 lactylation disrupts the PTBP1–TRIM21 association, thus blocking its ubiquitin–proteasomal degradation. These events, together with the downregulation of the de-lactylase SIRT1, give rise to a feed-forward loop which sustains malignant progression. (**B**) **SUMOylation.** The SUMOylation of PTBP2 is stable in normal brain but is decreased in glioma. It is hypothesized that this reduction may impair PTBP2 accumulation and weaken its antagonism toward PTBP1. Alongside miR-124 downregulation (which prevents the physiological PTBP1-to-PTBP2 switch), this contributes to PTBP1 pathway overactivation, though direct experimental validation is still required. (**C**) **Ubiquitination and deubiquitination.** Lactylated PTBP1 evades TRIM21-mediated degradation. In parallel, PTBP1 promotes exon 15 skipping in USP5 to generate an oncogenic isoform 2. All of these interrelated processes contribute to the proliferation and the migration of glioma cells. (Arrow notation: ➔, validated activation; ⊣, validated inhibition; ?, proposed relationship.).

Post-translational modifications (PTMs) also dynamically regulate PTBP1 function [64]. By integrating metabolic and signaling cues (Figure 3), these modifications precisely control PTBP1 stability and activity in response to cellular states, such as metabolic flux or pathway activation.

**Lactylation:** Lactylation promotes glioma malignancy by enhancing the RNA-binding capacity of PTBP1 and blocking its proteasomal degradation [25,65]. In glioma stem cells (GSCs), aberrant glycolysis drives lactate production, leading to high-level lactylation at PTBP1 lysine 436 (K436) [25]. This modification exerts dual effects on PTBP1: on one hand, it increases PTBP1 binding affinity for the 3′UTR of the downstream target, PFKFB4 mRNA, protecting PFKFB4 mRNA from nonsense-mediated decay and upregulating its expression. This drives glycolysis and promotes GSC proliferation, self-renewal, and invasion. On the other hand, K436 lactylation weakens PTBP1 interaction with the E3 ubiquitin ligase tripartite motif-containing protein 21 (TRIM21), suppressing ubiquitin–proteasomal degradation and enhancing PTBP1 stability [25] (Figure 3A). This further reinforces downstream target regulation and amplifies GSC proliferation.

In GSCs, the marked downregulation of the de-lactylase Sirtuin 1 (SIRT1) impairs PTBP1 de-lactylation, effectively locking the protein in its active, lactylated state [25]. This dual boost in RNA-binding affinity and protein stability, combined with diminished de-lactylase activity, robustly fortifies GSC glycolysis and stemness [25]. Ultimately, glycolysis-derived lactate feeds back to further promote PTBP1 lactylation, creating a self-reinforcing cycle that sustains glioma progression.

**SUMOylation:** Both PTBP1 and PTBP2 are substrates for SUMOylation; however, current evidence suggests that SUMOylation preferentially governs PTBP2 stability. In T98G glioma cells, PTBP2 SUMOylation is markedly lower than in normal brain tissue [66]. It has been hypothesized that this dysregulation may impair the physiological PTBP1-to-PTBP2 switch in glioma, which may result in persistent PTBP1 pathway activation and contribute to tumor progression [66] (Figure 3B). Because SUMOylation can regulate protein stability, reduced PTBP2 SUMOylation may impair PTBP2 accumulation and thereby weaken its antagonistic function toward PTBP1. However, direct experimental evidence that SUMOylation controls PTBP2 stability and PTBP1 antagonism in glioma is currently lacking, and this possibility should be regarded as a hypothesis. In addition, miR-124 downregulation in glioma also blocks the normal PTBP1-to-PTBP2 switching program [18,61]. Whether these two events cooperatively drive PTBP1 pathway overactivation in glioma remains to be experimentally tested.

**Ubiquitination and Deubiquitination:** The ubiquitination–deubiquitination equilibrium dictates PTBP1 stability and reshapes its downstream signaling network, ultimately determining glioma malignancy [67]. The E3 ubiquitin ligase TRIM21 directly binds to the RRM2 and RRM3 domains of PTBP1, promoting its ubiquitin–proteasomal degradation [25]. However, in GSCs, PTBP1 lactylation impedes this interaction, shielding the protein from ubiquitin-mediated degradation and ultimately leading to its accumulation for the purpose of fueling proliferation of the tumor cells [25]. Inhibiting PTBP1 lactylation in GSCs and orthotopic xenograft models restores ubiquitination-mediated degradation, thereby curbing stem cell self-renewal, proliferation, and invasion [25]. In turn, PTBP1 also controls alternative splicing of the deubiquitinase USP5. In U251 and LN229 glioma cells, high expression of PTBP1 drives exon 15 skipping within the USP5 transcript, generating a truncated isoform 2 that lacks 69 nucleotides and displays reduced deubiquitinase activity. When USP5 isoform 1 is forced to be expressed in these tumor cells, it dramatically inhibits proliferation and migration of the cells [53] (Figure 3C). These results reveal a bidirectional regulatory circuit: the ubiquitination machinery controls PTBP1 stability via TRIM21, while PTBP1 reciprocally dictates USP5 splicing. This reciprocal axis actively drives GSC maintenance and tumor progression.

## 5. PTBP1-Mediated Downstream Regulatory Mechanisms

**Figure 4:**
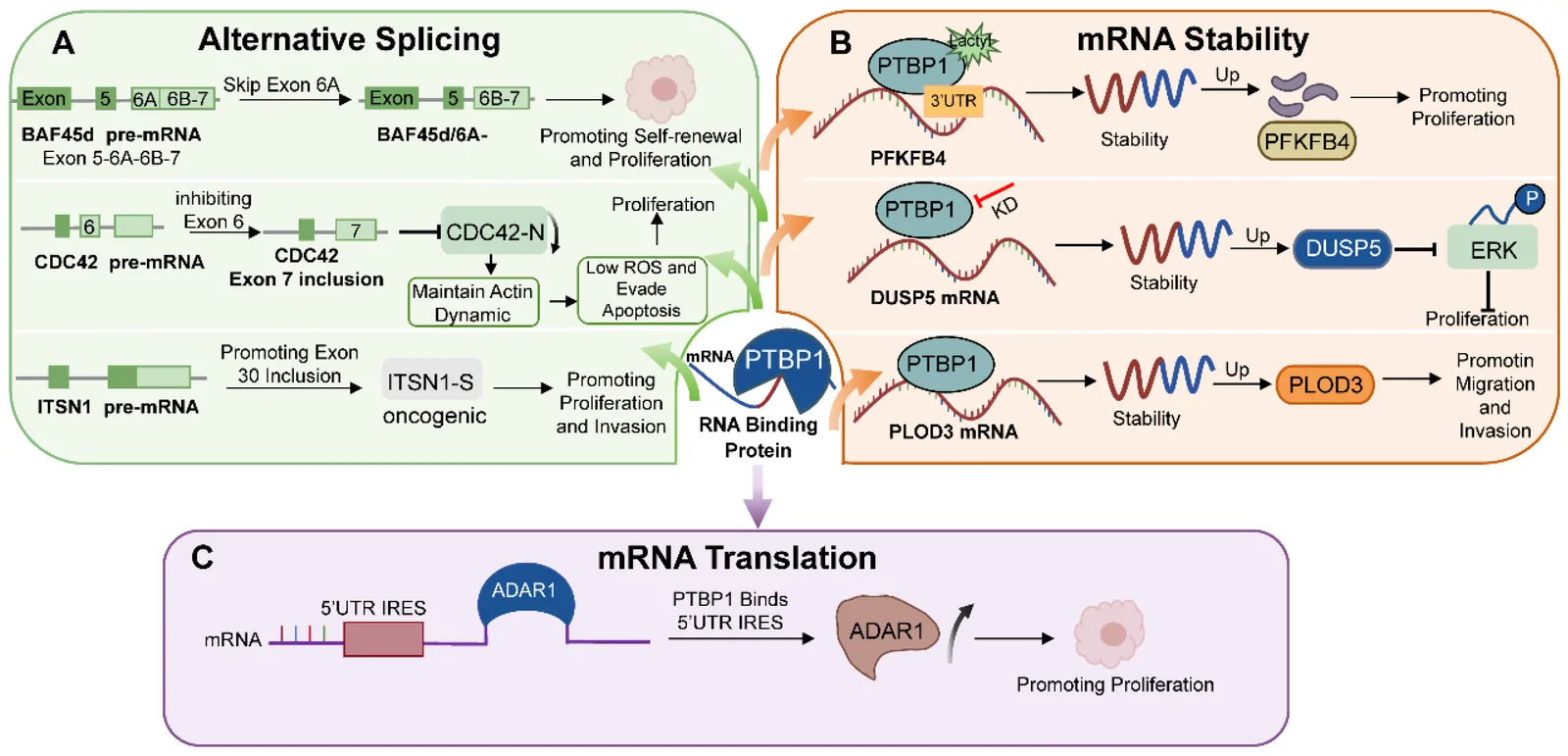
PTBP1 regulates post-transcriptional networks in high-grade glioma. (**A**) **Alternative splicing.** PTBP1 engages pyrimidine-rich motifs on target pre-mRNAs to dictate alternative splicing of BAF45d (exon 6A skipping), CDC42 (exon 6 skipping with favored exon 7 inclusion), and ITSN1 (exon 30 inclusion), yielding pro-proliferative and pro-invasive isoforms. (**B**) **mRNA stability.** PTBP1 directly binds and stabilizes PFKFB4 and PLOD3 transcripts to fuel proliferation, invasion, and migration. Conversely, PTBP1 knockdown (KD) stabilizes the tumor suppressor DUSP5, which in turn inhibits ERK1/2 phosphorylation and curtails glioma growth. (**C**) **Translational control.** PTBP1 acts as an IRES trans-acting factor, binding the ADAR1 mRNA 5′UTR to drive cap-independent translation of the ADAR1 p110 isoform and sustain tumor cell proliferation. Solid arrows (➔) indicate experimentally validated direct promotion or activation; solid T-bar lines (⊣) indicate experimentally validated direct inhibition or repression.

In high-grade gliomas, PTBP1 promotes tumor progression and therapy resistance via alternative splicing, mRNA stability, and translational control (Figure 4).

**Alternative Splicing:** Alternative splicing generates multiple mRNA isoforms through selective exon inclusion and is a key mechanism of eukaryotic gene regulation [68,69]. Its dysregulation is common in high-grade gliomas and drives malignant progression [70,71,72]. In high-grade gliomas, PTBP1 directly binds pyrimidine-rich sequences on pre-mRNAs to regulate alternative splicing of downstream targets, ultimately driving tumor progression [73]. For instance, Aldave et al. used global splicing array analysis to show that PTBP1 mediates skipping of BAF45d exon 6A in high-grade glioma tissues, generating a BAF45d/6A^−^ isoform. Silencing BAF45d/6A^−^ or overexpressing the exon-retaining BAF45d/6A^+^ isoform suppressed cell self-renewal and proliferation in patient-derived GSC23 and GSC11, and prolonged survival in an orthotopic xenograft model [26]. Yang et al. reported that in IDH-wt GBM, PTBP1 binds splicing silencer elements flanking exon 6 of the cell division cycle 42 (CDC42) pre-mRNA. By repressing neuron-specific exon inclusion (favoring exon 7), PTBP1 blocks the generation of the tumor-suppressive CDC42-N isoform. Restoring CDC42-N (or depleting PTBP1) disrupts actin cytoskeleton dynamics, triggering ROS accumulation and apoptosis. Conversely, by suppressing this isoform, PTBP1 preserves cytoskeletal integrity and low ROS levels, enabling glioma cells to evade apoptosis and sustain proliferation [74]. Lan et al. demonstrated that in LN229, U87MG, and primary glioma cells 140, PTBP1 directly binds a pyrimidine-rich sequence within ITSN1 exon 30, promoting exon inclusion and increasing the ratio of the oncogenic isoform ITSN1-S to the tumor-suppressive isoform ITSN1-L, thereby facilitating glioma proliferation and invasion [27]. Collectively, these studies show that PTBP1 drives glioma cell proliferation and invasion by targeting alternative splicing of key genes such as BAF45d, USP5, and ITSN1. This regulatory mechanism represents a crucial driving mechanism in glioma malignant progression (Figure 4A).

**mRNA Stability:** mRNA stability is a critical step in post-transcriptional gene regulation in eukaryotic cells, directly affecting protein abundance and biological function [75,76,77,78]. By directly binding and stabilizing downstream target mRNAs, PTBP1 reinforces stemness and drives glioma proliferation and EMT. Zhou et al. demonstrated that in patient-derived glioma stem cells (BT67, XT19), PTBP1 lactylation enhances its binding to the PFKFB4 mRNA 3′untranslated region (3′UTR), leading to a weakened interaction between PFKFB4 mRNA and the RNA helicase UPF1. This protects PFKFB4 mRNA from degradation and upregulates PFKFB4 protein expression. As a key regulatory enzyme in the glycolytic pathway, PFKFB4 controls glycolytic flux by synthesizing fructose-2,6-bisphosphate, an allosteric activator of phosphofructokinase-1 [79,80,81]. Thus, PFKFB4 overexpression drives aerobic glycolysis and promotes GSCs’ self-renewal and proliferation [25]. Using RNA immunoprecipitation, Li et al. confirmed that PTBP1 directly binds DUSP5 mRNA in GSCs, while PTBP1 knockdown enhances DUSP5 mRNA stability and upregulates its protein levels. This inhibits ERK1/2 phosphorylation, leading to neuronal-like differentiation, attenuating cell proliferation, reducing tumor volume and prolonging survival in an orthotopic xenograft model [54]. Additionally, Liu et al. found that in multiple glioblastoma cell lines (U118, U251), PTBP1 directly binds PLOD3 mRNA, enhancing its stability and upregulating PLOD3 expression to promote EMT, cell migration, and invasion [55]. Together, these findings establish PTBP1 as a key post-transcriptional regulatory node in glioma progression. By stabilizing mRNAs of targets such as PFKFB4 and PLOD3 and suppressing DUSP5, PTBP1 modulates tumor cell proliferation, differentiation, and EMT (Figure 4B). Interestingly, PTBP1 differentially regulates these targets by stabilizing PFKFB4 and PLOD3 while repressing DUSP5, underscoring its context-dependent function.

**mRNA Translation:** Malignant progression in high-grade gliomas is sustained by the selective activation of IRES-mediated cap-independent translation [82,83,84]. By binding to IRES elements within the 5′UTRs of target mRNAs, PTBP1 drives cap-independent translation and fuels malignant phenotypes [85]. In T98G, U87MG, and A172 glioma cells, the ADAR1 p110 protein accumulates robustly, whereas its mRNA levels remain lower than in normal astrocytes, indicative of translational deregulation. Mechanistically, PTBP1 directly binds an IRES-like element within the ADAR1 5′UTR to drive cap-independent translation of the p110 isoform. Knockdown of PTBP1 reduces ADAR1 p110 protein levels and IRES activity, thereby inhibiting glioma cell proliferation. Deletion of the IRES-like element abolishes ADAR1 p110 expression and fails to rescue the proliferation inhibition caused by PTBP1 knockdown [85], suggesting that ADAR1 p110 is a key effector of PTBP1′s pro-proliferative function. Collectively, PTBP1 drives glioma progression through IRES-dependent translational control of ADAR1 p110 (Figure 4C).

In high-grade gliomas, PTBP1 orchestrates malignant progression through a multilayered post-transcriptional program. It dictates pro-oncogenic splicing (BAF45d, USP5, ITSN1), stabilizes PFKFB4 and PLOD3 mRNAs, and suppresses DUSP5 to maintain ERK1/2 activation and an undifferentiated state. Beyond RNA processing, PTBP1 acts as an IRES trans-acting factor, driving cap-independent translation of targets such as ADAR1 p110. Collectively, these functions cement its role as a central regulatory hub in glioma.

## 6. Small-Molecule Drugs Targeting PTBP1: Current Preclinical Evidence

PTBP1 is aberrantly overexpressed in IDH-wildtype glioblastoma and portends poor overall survival, supporting its evaluation as a candidate prognostic biomarker and therapeutic target [24]. Consequently, several small-molecule compounds targeting PTBP1 have been identified. Therapeutic targeting of PTBP1 can be achieved via three distinct routes: direct binding, indirect modulation of the upstream regulators, or undefined downregulation. This review highlights three representative candidates, including venetoclax, PPM, and PT109, which exemplify direct binding, upstream modulation, and undefined downregulation, respectively.

**Small-molecule drugs directly targeting PTBP1:** Venetoclax was identified as an interacting compound by performing structure-based virtual screening using >7800 approved drugs and clinical compounds against the β-sheet scaffold of the RNA recognition motifs of PTBP1. Surface plasmon resonance (SPR) assays confirmed that venetoclax directly binds to PTBP1 with an equilibrium dissociation constant (Kd) of 7.61 μM. Although molecular docking suggests potential competition with RNA binding, direct RNA competition assays have not been reported. While venetoclax is an established BCL-2 inhibitor used clinically for hematological malignancies such as chronic lymphocytic leukemia and acute myeloid leukemia [86,87], its anti-proliferative efficacy in glioma stem cells (IC50 = 4.91μM) was significantly attenuated upon PTBP1 knockdown, indicating that its maximal antitumor effect is PTBP1-dependent [54]. However, this concentration is substantially higher than its sub-nanomolar potency against BCL-2, and venetoclax is approximately 99.9% plasma protein-bound and a P-glycoprotein substrate. Therefore, venetoclax is currently regarded as a repurposing candidate with a structure-based rationale and an unresolved mechanism, rather than a validated, selective PTBP1 inhibitor. In glioma cells, venetoclax exhibited a substantially lower IC50 than the first-line chemotherapeutic agent temozolomide (IC50 = 401.1μM), indicating more potent in vitro inhibition. However, systemic administration of free venetoclax failed to suppress glioma growth in vivo, likely due to limited blood–brain barrier (BBB) penetration. To overcome this, an Angiopep-2-modified PLGA nanoparticle (A2-PLGA/venetoclax) was developed. Intravenous administration of this nanoformulation (4 mg/kg on days 16, 18, and 20) improved brain exposure, significantly reduced tumor burden, and prolonged survival in orthotopic xenograft models with acceptable tolerability [54]. Beyond small molecules, nucleic acid-based nanotherapeutics targeting the PTBP1 axis are also emerging. An example of a nanoplatform based on liposomes loaded with shRNA against circ_0003137, an upstream PTBP1-interacting RNA, has been demonstrated to successfully cross the BBB and to exert a strong inhibition of tumor progression and improvement of prognosis in an intracranial glioblastoma model [55]. These results underscore the value of coupling PTBP1/PLOD3 axis inhibition with advanced nano-delivery systems to bypass the pharmacokinetic limitations of glioma therapy.

**Drugs acting indirectly by modulating upstream regulators of PTBP1:** In addition to directly binding to PTBP1, there may be other ways to inhibit its functions. For instance, 1,4,5,6,7,8-hexahydropyrido[4,3-d]pyrimidine (PPM) upregulates miR-873-3p to indirectly downregulate PTBP1. Through this axis, PPM suppresses PI3K/AKT/mTOR signaling, driving apoptosis and curbing glioma cell proliferation, migration, and invasion in preclinical models [88].

**Compounds that downregulate PTBP1 through undefined mechanisms:** The multi-kinase inhibitor PT109 has been shown to downregulate PTBP1, driving an increased pyruvate kinase M1 (PKM1) to pyruvate kinase M2 (PKM2) ratio and reprogramming glioblastoma cells toward an oligodendrocyte-like phenotype. Ultimately, this phenotypic shift attenuates tumor growth in preclinical models [89]. However, whether PT109 directly binds PTBP1 has not been established and its exact mechanism remains unclear.

In conclusion, PTBP1 represents both a candidate prognostic marker and a promising therapeutic target in high-grade gliomas. Although small-molecule inhibitors have shown preliminary antitumor activity in preclinical models, a few important points have to be addressed for clinical translation. Firstly, direct target engagement of candidate compounds in glioma cells has not been rigorously proven. Secondly, the majority of the antitumor evidence is derived from cell-based and/or limited xenograft models, and there is a lack of in vivo pharmacodynamic and toxicity data. Thirdly, BBB penetration is still a big hurdle to overcome for intracranial application. Fourthly, there is no PTBP1-targeting agent yet to enter clinical trials for glioma. Thus, advancing PTBP1 as a clinical target will involve robust target engagement, the use of several pharmacologically relevant in vivo models to assess efficacy, and the characterization of compound-specific BBB penetration and early-phase clinical trials.

## 7. Conclusions and Perspectives

The pathogenic role of PTBP1 in high-grade gliomas reveals the link between RNA-binding protein structural plasticity and post-transcriptional regulation. PTBP1 contains four RRMs, connected by flexible linkers. These RRMs recognize pyrimidine-rich pre-mRNA motifs in multiple combinatorial modes and form variable protein–protein interaction interfaces. This structural plasticity enables PTBP1 to participate in alternative splicing, mRNA stability and translational control. In gliomas, this structural framework is disrupted by dysregulated upstream signals, such as loss of miR-124, lactylation and non-coding RNA-mediated inhibition. Therefore, under certain circumstances, PTBP1 switches functions from providing physiological neuronal differentiation to a mainly oncogenic status to maintain stemness and therapy resistance. PTBP1 promotes glioma malignancy through three related post-transcriptional layers involving splicing regulation (BAF45d, USP5, ITSN1), mRNA stability modulation (stabilization of PFKFB4 and PLOD3, destabilization of DUSP5) and IRES-dependent translation (ADAR1 p110). These activities collectively contribute to metabolic reprogramming, apoptosis resistance, and increased invasive/migratory capabilities of glioma cells (Table 3).

Evidence levels: direct experimental intervention on the target molecule.

While PTBP1 is a promising target for therapy, there are a number of hurdles. Firstly, PTBP1 is involved in the development of normal neural cells [90,91] and the homeostasis of hematopoietic stem cells [92]. It is likely that complete inhibition can cause intolerable toxicity. Efforts should now focus on function-selective inhibitors that would interfere with oncogenic interactions without destroying the physiological functions. Secondly, compound-specific pharmacokinetic challenges limit the clinical translation of these compounds. Although venetoclax is able to penetrate the BBB, the exposure is highly limited owing to its high molecular weight and recognition as a substrate for efflux transporters (e.g., P-gp and BCRP), which leads to a high plasma-to-CSF ratio of 385:1. This limitation prompted the development of A2-PLGA/venetoclax nanoparticles [54,86]. Conversely, PT109 has achieved good BBB permeability with preclinical models (brain-to-plasma ratio 0.38) [93], but its effectiveness and target engagement need more in-depth exploration in glioma-specific models. Lastly, while the aforementioned mechanisms are largely cell-intrinsic, emerging evidence indicates that PTBP1 function is affected by the tumor microenvironment. For example, a single study has demonstrated that tumor-associated macrophage (TAM)-derived extracellular vesicles (EVs) deliver circ_0003137 to activate the PTBP1/PLOD3 axis [55]. Little is known, however, about the function and role of PTBP1 in other microenvironmental compartments (e.g., vascular endothelial cells, infiltrating lymphoid cells), hindering the rational design of combination targeting strategies.

Due to the structural and functional complexity of PTBP1, as well as longstanding obstacles in the study of its translation, we discuss a few future research avenues. Significantly, these approaches are still predominantly hypothesis-generating and must be carefully evaluated through experimental means before clinical translation. First, structural insights into PTBP1 RRM–RNA complexes offer a rational template for engineering allosteric inhibitors that target specific interaction interfaces rather than globally blocking RNA binding. Such interface-selective compounds could theoretically preserve physiological PTBP1 functions and reduce off-target toxicity. However, this approach remains limited by the lack of reported allosteric inhibitors and uncertain druggability of the RRM domains. Advancing this strategy will necessitate fragment-based screening, structure–activity relationship optimization, and rigorous target-engagement assays in glioma cells. Second, PTBP1 contains multiple surface-exposed lysine residues, including the lactylation-sensitive K436, and binds E3 ligases such as TRIM21, suggesting that PTBP1 is in principle amenable to targeted degradation. Proteolysis-targeting chimeras (PROTACs) could potentially achieve PTBP1-selective degradation and improve potency relative to occupancy-based inhibitors. However, no PTBP1-specific PROTAC has been reported, and achieving brain penetration with PROTACs remains a major challenge. Third, validation of PTBP1 protein levels and splicing products (e.g., the BAF45d/6A^−^ ratio) as candidate prognostic or chemoresponse biomarkers necessitates prospective, multicenter studies with standardized detection and pre-specified cutoff values. Fourth, indirect intervention strategies are conceptually attractive. Restoring the double-negative feedback loop between miR-124 and PTBP1 could dismantle PTBP1 expression while driving neuronal differentiation. Similarly, activating SIRT1 offers a theoretical approach to counteract PTBP1 lactylation and reverse its lactylation-driven stabilization. This rationale is mechanistically grounded in the observation that SIRT1 is downregulated in GSCs and that K436 lactylation directly enhances PTBP1 stability [25]. Translating these approaches to the clinic, however, presents distinct hurdles. miRNA-based therapies should achieve efficient BBB penetration while mitigating off-target and immune-related effects. In contrast, SIRT1 activators are constrained by their broad substrate specificity and potential pleiotropic effects. Overcoming these hurdles will necessitate the optimization of lipid nanoparticle or AAV delivery systems, the development of isoform-selective SIRT1 activators, and the validation of pharmacodynamic markers for PTBP1 de-lactylation. Finally, the role of PTBP1 in the glioma immune microenvironment remains largely undefined. Consequently, combining PTBP1 inhibition with immune checkpoint blockade is currently speculative. The rational design of such combinatorial strategies will necessitate single-cell and spatial transcriptomic profiling to map PTBP1’s function across tumor-associated macrophages, microglia, and T cells, a prerequisite for future clinical translation.

PTBP1 is a central hub linking structural plasticity to post-transcriptional regulation in high-grade gliomas. It also serves as a candidate prognostic marker and a therapeutic target with translational potential. Establishing PTBP1 as a clinically useful prognostic biomarker will require prospective, multicenter studies with standardized assays and pre-specified cutoff values. Although target specificity, brain delivery, and tumor heterogeneity remain substantial barriers, advances in structural biology, nanomedicine, and precision medicine offer promising avenues to refine PTBP1-targeted therapies. Once rigorously validated clinically, these strategies hold the potential to translate into tangible benefits for patients with high-grade gliomas.

## Figures and Tables

**Table 1:** Expression and mechanisms of action of PTBP1 in different tumor types.

Tumor Type	Expression	Key Mechanisms	Functional Outcomes	Evidence	Ref
Glioma	Overexpressed	Splicing regulation: PKM (PKM2↑)-IDH wt, ANXA7 (exon repression) -IDH wt, ITSN1 (exon 30 inclusion) -IDH wt, BAF45d (aberrant splicing) -IDH wt, CDC42 (neuron-specific exon repression)-IDH wt. Metabolic: stabilizes PFKFB4 mRNA- unstratified; interacts with HOTAIRM1 to stabilize SHMT2-IDH wt. Stemness: upregulates UNC5B upon knockdown-IDH wt. Post-translational: hyperlactylation (K436) inhibits degradation-unstratified.	Proliferation Invasion Stemness maintenance Therapy resistance Glycolytic reprogramming	Minigene assay, RIP, RNA pull-down, IP	[25,26,27,28,29,30,31,32]
Gastric cancer	Overexpressed	PTBP1 directly interacts with c-Myc and stabilizes it by preventing proteasomal degradation. Promotes PKM splicing (PKM2↑).	Enhanced glycolysis Proliferation Stemness maintenance	Co-IP, GST pull-down,	[21,33]
Colorectal cancer	Overexpressed	lncRNA FAM83H-AS1 (m^6^A-modified) interacts with PTBP1 RRM2 domain. PTBP1 crotonylation at K266 enhances hnRNP interaction to promote PKM2 splicing.	Chemo-resistance Proliferation	RIP, RNA pull-down, IP	[20,34]
Hepatocellular carcinoma	Upregulated	lncRNA IGFRIL acts as scaffold recruiting PTBP1 to destabilize IGFBP3 mRNA, activating IGF1R-AKT-mTOR axis. PTBP1 knockdown promotes ferroptosis via NCOA4 (context-dependent).	Pathway hyperactivation Tumor progression	RIP, RNA pull-down	[35,36]
Triple-negative breast cancer	Overexpressed	SLC7A5↑ → miR-152↓ → E2F1↑ → PTBP1 transcription↑ → PKM2↑ (PKM1-to-PKM2 switch).	Proliferation Therapy resistance	ChIP, Luciferase reporter	[37,38]
Bladder cancer	Overexpressed	circRNA biogenesis: binds ATIC pre-mRNA to promote circATIC synthesis. mRNA stability: circATIC sponges miR-1247-5p and recruits LIN28A to stabilize RCC2 mRNA, activating JNK/EMT.	Proliferation Metastasis Cisplatin resistance	ChIRP, RIP, Luciferase reporter	[39]

**Table 2:** Summary of non-coding RNAs regulating PTBP1 in glioma.

Category	Representative ncRNA	Mechanism of Regulation	Effect on PTBP1	Functional Consequence in Glioma	Evidence	Ref.
MicroRNA	miR-124 (IDH-wt)	Directly targets PTBP1 3’UTR; forms reciprocal repression loop with PTBP1	Downregulation	Promotes neuronal differentiation; locked in PTBP1-high/miR-124-low state in glioma	Luciferase reporter	[28,56]
	miR-128-1-5p (IDH-wt)	Directly targets PTBP1; sponged by lncRNA PVT1	Downregulation (when active)	PVT1 knockdown relieves miR-128-1-5p to suppress PTBP1, inhibit proliferation, and induce apoptosis	Luciferase reporter	[57]
	miR-760 (IDH-wt)	Directly targets PTBP1; sponged by circYIPF6	Downregulation (when active)	circYIPF6 upregulation sequesters miR-760, leading to PTBP1 overexpression, enhanced proliferation and glycolysis, reduced apoptosis	Luciferase reporter	[58]
	miR-9 (IDH-wt)	PTB-AS masks miR-9 binding site in PTBP1 3’UTR	Stabilization (prevents miR-9-mediated repression)	Contributes to PTBP1 stabilization and oncogenic function	Luciferase reporter, RIP	[29]
Long non-coding RNA	PTB-AS (IDH-wt)	Binds to PTBP1 3’UTR, stabilizes mRNA, masks miR-9 sites; cooperates with SND1	Upregulation (stability)	Knockdown reduces PTBP1, inhibits proliferation, migration, and tumor growth	RIP, RNA pull-down	[29]
	ST7-AS1 (IDH-wt)	Binds PTBP1 and promotes its downregulation (tumor suppressor)	Downregulation	Overexpression inhibits glioma progression via Wnt/β-catenin suppression	RIP, RNA pull-down	[59]
	HOTAIRM1 (IDH-wt)	Recruits PTBP1 and IGF2BP2 to SHMT2 mRNA, enhancing stability	Functional cooperation	Maintains serine metabolism and mitochondrial function; targeting HOTAIRM1 disrupts these processes	RIP, RNA pull-down	[31]
Circular RNA	circYIPF6 (IDH-wt)	Sponges miR-760, derepressing PTBP1	Upregulation	Promotes proliferation, glycolysis, suppresses apoptosis; knockdown inhibits glioma growth	correlation	[58]
	circSMARCA5 (IDH-wt)	Binds SRSF1, alters SRSF3 pre-mRNA splicing, indirectly regulating PTBP1	Indirect downregulation	Inhibits cells migration	eCLIP, RNA pull-down	[60]
	circ_0003137 (IDH-wt)	Directly binds PTBP1 protein; enhances PLOD3 mRNA stability.	Functional interaction (stabilizes target mRNA)	Promotes EMT, migration, invasion	RIP, RNA pull-down	[55]

**Table 3:** Evidence levels for PTBP1-related mechanisms in glioma.

Mechanism/Target	Cell Line	Patient-Derived GSC	Animal Model	Clinical Specimen	Clinical Trial	Key Limitation	Ref.
BAF45d exon 6A skipping	√	√	√	√	-	Limited in vivo validation	[26]
CDC42 exon 6 repression	√	-	-	±	-	Limited in vivo validation	[74]
ITSN1 exon 30 inclusion	√	√	-	√	-	No orthotopic model	[27]
USP5 exon 15 skipping	√	-	-	-	-	Cell-line only	[53]
PFKFB4 mRNA stabilization	√	√	√	-	-	GSC/orthotopic only	[25]
DUSP5 suppression	√	√	√	-	-	limited clinical data	[54]
PLOD3 mRNA stabilization	√	-	-	-	-	Cell-line only	[55]
ADAR1 p110 IRES translation	√	-	-	±	-	No in vivo validation	[85]
miR-124/PTBP1 switch	√	±	√	√	-	Context-dependent	[56]
K436 lactylation	√	√	√	-	-	No clinical validation	[25]
Venetoclax	√	√	√	-	-	BCL-2 contribution not excluded	[54]
PT109	√	-	±	-	-	No direct PTBP1 engagement	[89]

(√, direct experimental evidence; ±, partial or indirect evidence; -, no reported evidence. “Clinical specimen” refers to human glioma tissue-based analyses; “clinical trial” refers to prospective interventional studies).

## Data Availability

No new data were created or analyzed in this study. Data sharing is not applicable to this article.

## Abbreviations

A2-PLGA	Angiopep-2-modified poly (lactic-co-glycolic acid)
ADAR1	Adenosine deaminase RNA-specific 1
AML	Acute myeloid leukemia
ATG5	Autophagy-related 5
BAF45d	Brahma/SWI2-related gene 1 (BRG1)-associated factor 45d
BBB	Blood–brain barrier
BCL-2	B-cell lymphoma 2
CAFs	Cancer-associated fibroblasts
CDC42	Cell division cycle 42
CDC42-G	General CDC42 isoform
CDC42-N	Neuron-specific CDC42 isoform
ceRNA	Competing endogenous RNA
ChIRP	Chromatin Isolation by RNA Purification
circRNAs	Circular RNAs
CLIP	Cross-linking and immunoprecipitation (also eCLIP enhanced CLIP)
CNS	Central nervous system
CSF	Cerebrospinal fluid
DUSP5	Dual-specificity phosphatase 5
EMT	Epithelial–mesenchymal transition
EVs	Extracellular vesicles
GBM	Glioblastoma
GSCs	Glioma stem cells
HGGs	High-grade gliomas
HIF1α	Hypoxia-inducible factor 1-alpha
IC50	Half-maximal inhibitory concentration
IDH	Isocitrate dehydrogenase
IDH-wt	IDH-wildtype
IDH-mut	IDH-mutant
IRES	Internal ribosome entry site
ITSN1	Intersectin 1
Kd	Equilibrium dissociation constant
KMT2A-r	KMT2A-rearranged
lncRNAs	Long non-coding RNAs
miRNAs	MicroRNAs
MSCs	Mesenchymal stem cells
mRNA	Messenger RNA
ncRNAs	Non-coding RNAs
NSCLC	Non-small-cell lung cancer
P-gp	P-glycoprotein
PFKFB4	6-phosphofructo-2-kinase/fructose-2,6-biphosphatase 4
PKM	Pyruvate kinase M (PKM1 pyruvate kinase M1; PKM2 pyruvate kinase M2)
PLGA	Poly (lactic-co-glycolic acid)
PLOD3	Procollagen-lysine, 2-oxoglutarate 5-dioxygenase 3
PPM	1,4,5,6,7,8-hexahydropyrido[4,3-d] pyrimidine
PRMT5	Protein arginine methyltransferase 5
PROTACs	Proteolysis-targeting chimeras
PTBP1	Polypyrimidine tract-binding protein 1
PTMs	Post-translational modifications
PVT1	Plasmacytoma variant translocation 1
RIP	RNA immunoprecipitation
ROS	Reactive oxygen species
RRMs	RNA recognition motifs
SIRT1	Sirtuin 1
SND1	Staphylococcal nuclease domain-containing 1
SPR	Surface plasmon resonance
SRSF1	Serine and arginine rich splicing factor 1
SRSF3	Serine and arginine rich splicing factor 3
ST7-AS1	ST7 antisense RNA 1
SUMO	Small ubiquitin-like modifier (SUMOylation)
TAMs	Tumor-associated macrophages
TRIM21	Tripartite motif-containing protein 21
USP5	Ubiquitin-specific peptidase 5
WHO	World Health Organization
